# Patient-derived pancreatic tumor bacteria exhibit oncogenic properties and are recognized by MAIT cells in tumor spheroids

**DOI:** 10.3389/fimmu.2025.1553034

**Published:** 2025-04-22

**Authors:** Poojabahen Tajpara, Michał Jacek Sobkowiak, Katie Healy, Sabrina Naud, Beate Gündel, Asif Halimi, Zara Ahmad Khan, Giorgio Gabarrini, Sylvie Le Guyader, Gabriela Imreh, Julie A. Reisz, Marco Del Chiaro, Angelo D’Alessandro, Rainer Heuchel, J Matthias Löhr, Volkan Özenci, Margaret Sällberg Chen

**Affiliations:** ^1^ Division of Pathology, Department of Laboratory Medicine, Karolinska Institutet, Huddinge, Sweden; ^2^ Division of Oral Diagnostics and Surgery, Department of Dental Medicine, Karolinska Institutet, Huddinge, Sweden; ^3^ Division of Clinical Microbiology, Department of Laboratory Medicine, Karolinska Institutet, Huddinge, Sweden; ^4^ Pancreas Cancer Research Lab, Department of Clinical Science, Intervention and Technology, Karolinska Institutet, Huddinge, Sweden; ^5^ Division of Surgery, Department of Clinical Science, Intervention and Technology, Karolinska Institutet, Stockholm, Sweden; ^6^ Department of Surgical and Perioperative Sciences, Surgery, Umeå University, Umea, Sweden; ^7^ Department of Medicine Huddinge, Karolinska Institutet, Huddinge, Sweden; ^8^ Department of Biochemistry and Molecular Genetics, University of Colorado, Aurora, CO, United States; ^9^ Division of Surgical Oncology, Department of Surgery, University of Colorado of Medicine, Aurora, CO, United States; ^10^ Department of Cellular Therapy and Allogeneic Stem Cell Transplantation (CAST), Karolinska University Hospital Huddinge and Karolinska Comprehensive Cancer Center, Stockholm, Sweden

**Keywords:** pancreatic neoplasm, tumor microbiota, metabolites, spheroids, DNA damage, MAIT cells, immunotherapy

## Abstract

**Introduction:**

Tumor-residing microbiota poses a new challenge in cancer progression and therapy; however, the functional behavior of patient tumor-derived microbes remains poorly understood. We previously reported the presence of tumor microbiota in intraductal papillary mucinous neoplasms (IPMNs), which are precursors of pancreatic cancer.

**Methods:**

We examined the metabolic and pathogenic potential of clinical microbiota strains obtained from IPMN tumors using various pancreatic cell lines and 3D spheroid models.

**Results:**

Our findings revealed that several strains from IPMNs with invasive cancer or high-grade dysplasia, such as *E. cloacae, E. faecalis*, and *K. pneumoniae*, induced a cancer metabolite signature in human pancreatic cells when infected *ex vivo*. Bacterial invasiveness was significantly correlated with DNA damage in spheroids derived from normal and tumor-derived pancreatic cells, particularly in strains derived from advanced neoplasia IPMN and under hypoxic conditions. Additionally, microbial metabolites activate human mucosal-associated invariant T (MAIT) cells and restrict the infection, both extra- and intracellularly, in hypoxic tumor conditions and in synergy with antibiotics.

**Discussion:**

Immune sensing of tumor microbiota metabolites may have clinical implications in cancer management.

## 1 Introduction

Pancreatic cancer (PC), characterized by a dismal 5-year relative survival rate of 8-12% (1), has become a focus of recent research examining the intratumoral microbiome’s impact on therapeutic outcomes and patient survival (2). The emerging “oncobiome” concept, encompassing the collective genomes of microorganisms linked to cancer, highlights the significance of understanding microbial influences in cancer development (3). This has extended to the microbiome within preneoplastic lesions, exploring complex interactions between microbial communities and the local tumor microenvironment (TME) (3). The TME is a complex and dynamic milieu surrounding cancer cells that plays a crucial role in cancer progression. During this progression, cancer and stromal cells have restricted access to oxygen due to the aberrant vascularization and a poor blood supply. Hypoxia is a crucial factor for bacterial adhesion and invasion mechanisms. While *Helicobacter pylori* and certain *Escherichia coli* bacteria strains show higher adaptation capabilities in hypoxic environment and associate with preneoplastic lesions preceding colorectal preneoplasia, the field is expanding rapidly to include the oncobiome and remains dynamic (4–7).

In our previous studies, we discovered that intraductal papillary mucinous neoplasms (IPMNs), cystic pancreatic tumors, and precursor lesions to PC harbor distinct tumor microbiota (6). This local microbiota has been found to correlate with elevated levels of inflammatory cytokines and microbial lipopolysaccharides in the advanced neoplastic stages (6, 8). In *ex vivo* experiments, we observed that live cultivated bacteria from IPMN can infect monolayers of both healthy pancreatic cells and cancer cells at different stages of differentiation with varying efficiencies. Notably, gamma-proteobacteria infections in monolayers of pancreatic cells induced the formation of γH2A.X, a DNA damage marker, and exhibited intracellular hiding capabilities that allowed them to avoid being killed by antibiotics as shown under experimental conditions (9). The metabolic signatures observed in pancreatic cyst fluids from IPMN patients resembled bacterial activities that involve lipopolysacharides, IL-1b, as well as microbiota-associated bile acids, trimethylamine-oxide, and polyamines like cadaverine, putrescine (6, 9, 10) The invasion of microbes into tumors has garnered significant interest, as it appears to play a crucial role in cancer initiation, progression, and therapeutic responses. Conversely, targeting the PC microbiota has the potential to enhance cancer immunotherapy and improve immunogenicity within the tumor microenvironment (11). However, further research is needed to investigate interactions between the microbiome, tumor cells, host immune system, and metabolic pathways involved.

Pancreatic cystic tumors, particularly IPMNs, have an increased risk of malignant transformation with a subset that will progress to invasive cancer, making them clinically significant. IPMNs are classified at different neoplastic levels, including low-grade dysplasia (LGD), high-grade dysplasia (HGD), and invasive cancer (C) (1). Understanding the role of microbiota in oncogenesis and immunomodulation in this disease is crucial. Recent evidence suggests that mucosal-associated invariant T (MAIT) cells, a conserved subset of T cells responsive to microbial metabolites, infiltrate pancreatic ductal adenocarcinoma (12–14). While studies on colorectal cancer demonstrated MAIT cell activation in response to tumor bacterial infection to laboratory strains of *Fusobacterium nucleatum (* 15), it remains unclear whether the MAIT cell sensing mechanism extends to clinical strains found in pancreatic tumors or if it senses pathogenic bacterial behaviors in the hypoxic tumor microenvironment.

In this study, we aimed to investigate whether live cultivated bacteria isolated from patient IPMNs with varying neoplastic grades can influence oncogenic pathways in pancreatic cells. To mimic the spatial environment of pancreatic tumors, we established new pancreatic spheroid models to mimic the TME and hypoxic influences. We hypothesized that IPMN tumor-derived bacteria would colonize pancreatic spheroids and induce oncogenic changes which in turn could be recognized and counteracted by MAIT cells. We employed methods ranging from 3D spheroid imaging, through microbial metabolism prediction analysis and various immunological methods to confirm our hypothesis.

## 2 Materials and methods

### 2.1 Microbial cultivation and species confirmation

Patient-derived bacterial species were isolated and cultured as previously described (9). Briefly, pure colonies obtained on agar were subjected to species identification using matrix-assisted laser desorption/ionization time-of-flight mass spectrometry (MALDI-TOF MS) before use. MALDI-TOF MS scores ≥1.70 and ≥2.00 were accepted as successful confirmation at the genus and species levels, respectively. Microbial cultivations were proceeded on blood agar and incubated under aerobic and anaerobic conditions at 37°C for 48 h. Anaerobic bacteria cultivations were performed using an anaerobic box with GasPak (ThermoFisher Scientific, Waltham, MA, USA). *Granulicatella adiacens* was grown on blood agar supplemented with 100 µL 0.01% pyridoxal hydrochloride (ThermoFisher Scientific). *E. cloacae* sensitivity to gentamicin was confirmed by MIC testing (Disk Diffusion Test) reference breakpoint <2 mg/L (16, 17).

### 2.2 Secretome preparation

Single colonies of identity-confirmed bacterial isolate strains were picked and cultured in 5mL of liquid BHI (Brain Heart Infusion) medium (Karolinska University Hospital, Huddinge, Sweden) for 24 hours at 37°C. The cultures were first centrifuged at 4000 RPM for 20 minutes at 4°C to exclude the pellet, and the supernatant was centrifuged at 1100 RPM for 5 minutes at 4°C. Supernatants were obtained from either bacterial culture or overnight co-cultures with pancreatic cells (5) and filtered twice through a 0.2 µm filter before being used for MAIT cell activation assays. *E. coli* was used as positive control, and the BHI medium as negative control (18).

### 2.3 Cell lines and donor PBMC

The immortalized pancreatic cell lines: normal pancreatic cells (hTERT-HPNE) or PC cells of early (Capan-2) and late (AsPC-1) differentiation stage, were maintained in a specialized medium formulated in accordance with the supplier’s specifications (9). Peripheral blood mononuclear cells (PBMCs) were isolated from fresh buffy coats obtained from healthy donor blood at Karolinska University Hospital, Huddinge, Sweden, using Ficoll-Paque density gradient separation. MAIT cells were isolated from fresh healthy donor peripheral blood mononuclear cells (PBMCs) and expanded as previously described (19, 20). The expansion of MAIT cells yielded high-purity cultures (**Supplementary Figure S5**). Isolated PBMCs and expanded MAIT cells were frozen in freezing medium (FCS in 10% DMSO) (FCS and DMSO, Sigma, St Louis, MO, USA) immediately after isolation/expansion and stored in liquid nitrogen until use.

### 2.4 Biological material and permissions

This study followed the Helsinki convention, and the collection and use of biological materials were approved by the Regional Ethics Committee of Stockholm (Dnr. 2015/1580-31/1 and Dnr 2007/115-31/1, Dnr 2024-04297-02).

### 2.5 Metabolomics analysis of supernatants

Supernatant samples were analyzed using mass spectrometry-based metabolomics at the University of Colorado School of Medicine Metabolomics Facility. Supernatant aliquots were thawed on ice then a 10 mL aliquot was diluted with 240 mL of cold 5:3:2 methanol:acetonitrile:water (v/v/v) and vortexed for 30 min at 4°C, as previously described (21). Supernatants were clarified (10,000 × g, 10 min, 4°C) and then analyzed via 20 mL injections on a Thermo Vanquish UHPLC coupled to a Thermo Q Exactive mass spectrometer operating in positive and negative ion modes (separate runs) with a 5 min C18 gradient, as described previously (22). Raw files were converted to mzXML using RawConverter, and metabolites were annotated and integrated in Maven using the KEGG database. Quality control assessment of instrument performance and peak annotation was performed as previously described (21).

### 2.6 Supernatant activation and flow cytometry

PBMCs isolated from healthy donor blood were resuspended at 2x10 cells/mL in R10 medium (RPMI-1640, Thermo Fisher Scientific +10% FCS, Sigma) as described earlier (6). After overnight rest (37°C, 5% CO_2_) the cells were washed once in PBS, and resuspended again at 2x10 cells/mL in R10 supplemented with 100 mg/mL normocin (InVivoGen, San Diego, CA, US). Cell suspension was then mixed with bacterial culture supernatant to final volume/volume ratio of 4:1 and cultivated further at 37°C, 5% CO_2_. After 18 hours, GolgiStop and GolgiPlug protein transport inhibitors (BD Biosciences, Franklin Lakes, NJ, USA) were added at the final dilution of 1:1000 and 1:500, respectively. After incubation for a total of 24 hours, cell suspensions were transferred to a fresh V-bottom plate and washed once with FACS buffer (PBS + 2% FCS + 2mM EDTA). Then stained with PE-conjugated MR1:5-OP-RU tetramer (NIH Tetramer Core Facility) (diluted 1:80 in FACS buffer) for 20 minutes at room temperature. Then washed once with FACS buffer and surface-stained at 4°C for 20 minutes with directly conjugated antibodies in FACS buffer: CD3-BV650, CD4-BV711, CD8α-BV570, CD39-BV605 (all from BioLegend, San Diego, CA, USA), CD69-ECD (Beckman Coulter, Indianapolis, IN, USA), and CD161-PE-Cy5 (BD Biosciences). Fixation was done in Transcription Factor Fixation/Permeabilization buffer (BD Biosciences) as per manufacturer’s instructions, followed by acquisition by the LSRFortessa 18-colour flow cytometer equipped with 405, 488, 561, and 639 nm lasers. Single-stained polystyrene beads were used for software-based compensation in the FlowJo v 10.2 software with gating strategy for MAIT cells (**Supplementary Figure 1**) as described earlier (23).

### 2.7 Spheroid preparation, infection and, multiplex cytokine analysis experiments

The hTERT-HPNE cells were trypsinized and seeded at 20,000 cells/well in a Nunclon sphere 96 well plate (Thermo Fisher Scientific) in 150ul medium as described above. Heterospheroids consisting of PANC1 cells and murine pancreatic stellate cells (mPSCs) (24) were cultured in DMEM (low glucose (1 g/L), 10% FBS) and prepared as described previously (25). All spheroids were preincubated at 37°C in normoxic (5% CO_2_, 20%O_2_) and hypoxic (5% CO_2_, 1% O_2_) incubators for 24 h prior to bacterial exposure. Bacteria were diluted in RPMI-1640 + 2% human serum (Sigma) (R2 medium) and added 150mL to spheroids at a final multiplicity of infection (MOI) of 1 for 18 h. For the MAIT cell coculture experiments, the bacteria and pancreas spheroids were first co-incubated for 6 h followed by PBS wash before the addition of either expanded MAIT cells (20,000/well) or Normocin (InVivoGen) (100mg/mL) for 18 h in 100mL volume of R2 medium. Supernatants were collected for the ProcartaPlex multiplex cytokine analysis of IFNg, TNFa, IL-2, IL-12, IL-1b, IL-4, IL-6, IL-10, IL-17A (CTLA-8, Thermo Fisher Scientific) according to manufacturer’s instruction. For intracellular infection, bacteria were added at MOI 10 for 6 h followed by PBS wash before addition of gentamicin (9) treatment for 1 h to kill extracellular bacteria, followed by PBS wash and then incubation with or without expanded MAIT cells (20,000/well), penicillin-streptomycin (100mg/mL), or a combination of the latter two, for another 18 h. The supernatant and lysed cell pellets were cultivated on blood agar plates, and the bacterial load was counted and expressed as colony forming units (CFU).

### 2.8 Immunostaining, mounting and image acquisition

Bacterial penetration in 3D spheroids was measured by labeling live bacteria with HADA (fluorescent D-amino acid, labeling peptidoglycans) (R&D Systems, Minneapolis, MN, US) and DRAQ7 (26). After 18h of exposure to the indicated strain, the spheroids were incubated for 4 h with either HADA or PBS (control). They were then washed twice in 100mL of phosphate buffer saline PBS, fixed with 100ul of 4% paraformaldehyde (Sigma) for 1h at room temperature, washed again with 100mL of PBS, and permeabilized in 0.5% triton x-100 (Sigma) for 45 min. To ensure the specificity of the HADA staining towards bacteria, the fixed spheroids were labelled with DAPI (Thermo Fisher Scientific) which identifies human nuclei, and DRAQ7 which binds to both human and bacterial DNA. The staining protocol developed from an earlier described method (27). Briefly, the spheroids were incubated at room temperature (RT) in anti-phospho-histone H2A.X antibody labelled with Alexa Fluor 488 (1:100 dilution) for 12h at RT, removed, and then incubated for another 4h in 100mL of DRAQ7 (1:100) and DAPI (1 mg/mL) containing 0.1% tween-20.

For spheroid mounting, clearing, and 3D light sheet imaging, a bed of 10% polyacrylamide gel (Invitrogen) was attached to a clean glass slide (Thermo Fisher Scientific) using 1.2% low-melting-point agarose (Sigma) in water. The spheroids were laid on top of the acrylamide bed and covered with agarose. The samples and mounting material were then passively cleared together at room temperature and in the dark for at least 96h by inserting the glass slide into a 50mL Falcon tube filled with 1 part cubic-2 (28) and 4 parts 85% glycerol (Thermo Fisher Scientific) (in water). Three hours prior to imaging, the samples were transferred to a new 85% glycerol solution (without cubic-2). The slide and spheroids were then placed at the bottom of the microscope imaging container, secured with putty, and covered with 85% glycerol. Images were acquired using an Aurora Airy beam light sheet microscope (MSquared lasers, UK) equipped with two Hamamatsu Orca Flash IV (6.5 mm pixels), a 7-line laser bed (Omicron Laserage, Germany), and immersion objectives (Navitar, USA) with variable numerical aperture, field of view, and magnification depending on the refractive index of the immersion medium. At the refractive index of 85% glycerol (1.449), the magnification and the numerical apertures were 16.649 and 0.391, respectively. The images were acquired sequentially using one laser (ex) and one emission filter (em) as follows: DAPI (405ex and 445/58em), Alexa Fluor 488 (488ex and 525/50em) and DRAQ7 (485ex and 667/30em). For each spheroid, a z-stack was acquired using a stage scanner with a z-step of 0.4 mm. Using these imaging settings, the Nyquist sampling criterion was fulfilled in the xy and z dimensions for all channels, with a slight under-sampling in xy in the blue channel. The laser power and exposure time for each channel were identical for all the samples and were set to avoid saturation. The images were deconvolved with MSquared Deconvolution software using the Richardson-Lucy algorithm (100 iterations, 16-bit, with chromatic and curvature corrections).

### 2.9 Oxidative DNA damage competitive ELISA

hTERT-HPNE and PANC-1 cells (50,000 cells/well) in flat bottom 96 well plate (Corning, NY, US) and hTERT-HPNE spheroids were maintained as mentioned above in hypoxic chamber. Following infection (MOI 1) by respective anaerobic bacteria in hypoxic condition, the culture supernatant was collected at 6 respectively 24 hours for measurement of oxidative DNA damage. Quantification of was performed with capture rabbit antibodies by ELISA, with standard curve generated by titration of 8-hydroxy-2’-deoxyguanosine, according to manufacturer’s instructions (DNA damage competitive ELISA, Thermo Fisher Scientific).

### 2.10 Image analysis and calculation index

The total volume of each spheroid was first estimated in the DAPI channel (surface tool function at smoothing 5.50 um) in the IMARIS software (version 9.9.0, Andor, UK). The DRAQ7 stained volume was then segmented (surface smoothing 7 um) and thereafter subtracted from DAPI fraction to estimate the bacteria-specific volume. The specificity was verified against staining with HADA in spheroids with or without infection (MOI 1) (**Supplementary Figures 2A, B**). Quantification of area classified as bacteria invasion or DNA damage acquired in the IMARIS software was collected from individual spheroids for subsequent data analysis (**Supplementary Figure 2C**). The relative volume of nuclei and DNA damage were measured from the Alexa Fluor 488 channel using the Spot tool (9 um diameter) with settings identified for individual nuclei.

## 3 Results

### 3.1 Patient-derived tumor bacteria strains cause oncometabolic disturbances in pancreatic cells and spheroids

**Figure 1:**
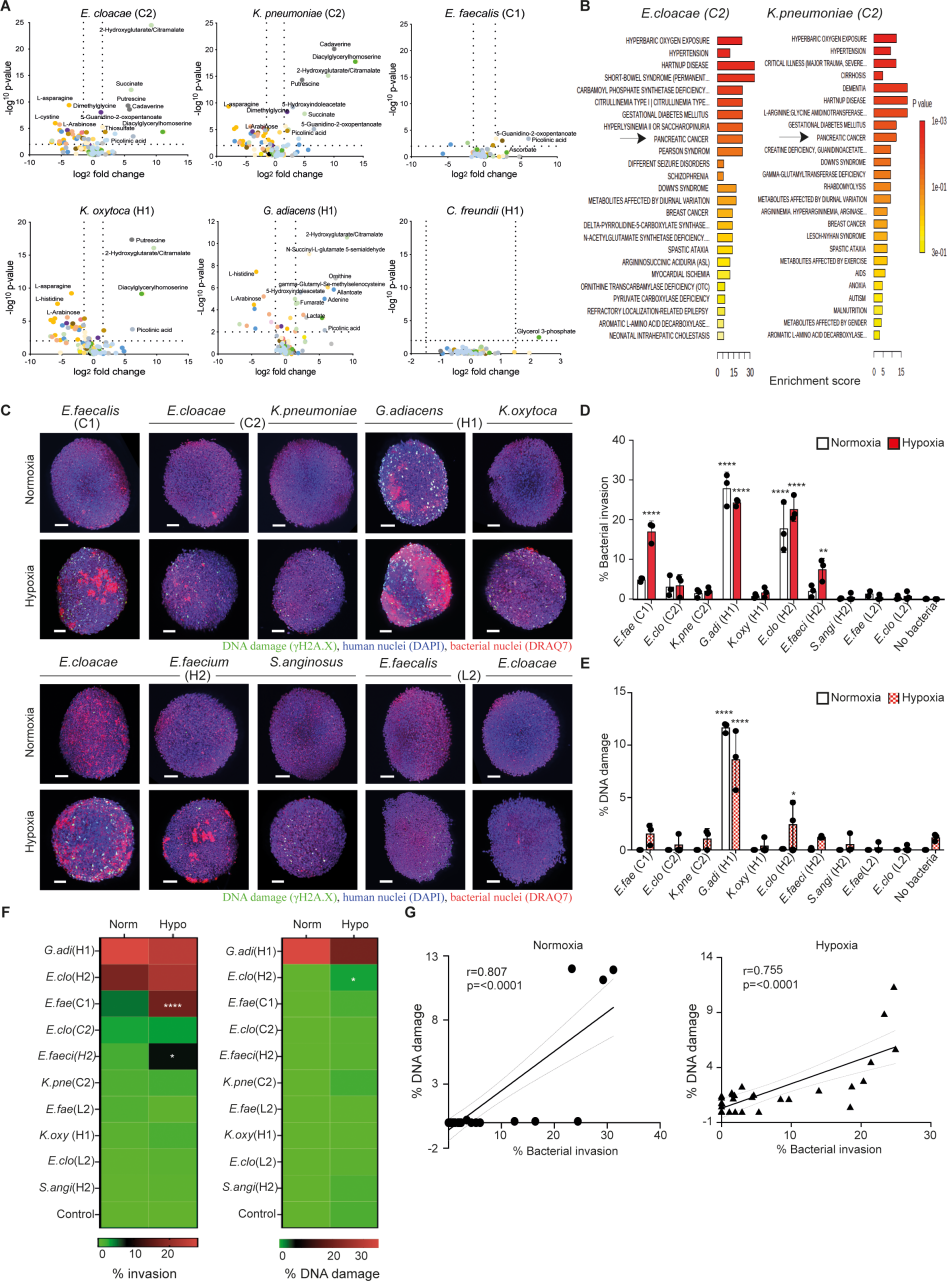
Patient-derived pancreas tumor bacteria induce metabolic aberrations and pathogenicity in pancreatic cells and spheroids. **(A)** Metabolomic profiling of bacteria-induced secretome in indicated bacteria-exposed pancreatic cells (hTERT-HPNE, AsPC-1, or Capan-2). The culture supernatants after overnight incubation were subjected to mass spectrometry-based metabolomics and the levels of identified compounds of exposed cells were compared against the unexposed controls. The results from the three cell types are combined and analyzed specifically to respective bacterium on Volcano plots by Log2 fold-change (X-axis) and inverted p-value (Y-axis). **(B)** MSEA (MetaboAnalyst 5.0) overrepresentation analysis against disease-associated blood metabolite set for indicated bacterial condition. **(C)** Representative images of hTERT-HPNE spheroids co-cultured with and without indicated IPMN-derived bacterial strains at MOI 1 under either normoxic or hypoxic condition after 18 h of exposure. Bacterial strains were isolated from IPMN tumor cases diagnosed with invasive cancer (C1 and C2), high-grade dysplasia (H1 and H2), or a low-grade dysplasia case (L2). Stained spheroids are visualized by the 3D light sheet imaging to measure phosphorylated γH2A.X formation (green, DNA damage), human nuclei (blue, DAPI), human and bacterial nuclei (red, DRAQ7). Scale bar = 100mm. **(D, E)** Summary of three experimental repeats of quantitative analysis of **(D)** bacterial invasion (percentage refers to the invasion of the spheroid as a whole) or **(E)** bacterial-associated DNA damage in hTERT-HPNE spheroids under either normoxia condition (white bars) or hypoxia condition (red bars). **(F)** Level of invasiveness of bacterial strains from high to low and significant differences between normoxia and hypoxia are indicated. **(G)** Correlation between bacterial invasion and DNA damage under normoxia or hypoxia. Two way-ANOVA was used for group comparisons. *p < 0.05, **p < 0.01, ****p < 0.0001.

Species identity of clinical strains obtained from IPMN cystic tumors (9) was confirmed using MALDI-TOF mass spectrometry. Based on the neoplastic level of their host, each strain was designated as L (low-grade dysplasia), H (high-grade dysplasia), or C (invasive cancer) (9) followed by the respective case numbers (**Supplementary Figure 3A** **).** Initially, we examined bacteria-induced metabolites in different pancreatic cell lines, hTERT-HPNE (normal human pancreatic duct epithelial cells) and PC cancer cell lines (AsPC-1 or Capan-2), using a high-throughput mass spectrometry-based metabolomics approach. We noticed that the profiles of activated metabolites from PC cells were quite similar among these pancreatic cell lines and, were specifically associated with the bacterial insult (**Supplementary Figure 3B** upper panel), as antibiotic treatment reduced the release of these compounds in all three cell types (**Supplementary Figure 3B** lower panel). Metabolite data of individual strains from those co-culture conditions are available in the BioStudies database (https://www.ebi.ac.uk/biostudies/) (29) under accession number S-BSST1747. Our results further indicate that among the different patient strains, Gammaproteobacterial species significantly increased the levels of cadaverine, putrescine, succinate, thiosulfate, N-Succinyl-L-glutamate, and diacylglycerylhomoserine in the culture supernatant (2-10e3 fold increase relative to unexposed controls, p<0.05) (**Figure 1A**). In contrast, Bacilli species induced lactate, fumarate, and 5-hydroxyindoleacetate (2-10e1 fold increase relative to unexposed controls, p<0.05), while 2-hydroxyglutarate/citramalate and picolinic acid were generally detected across bacteria exposed conditions. Notably, three of strains isolated from the invasive cancer or high-grade dysplasia cases (C2 or H2) - *E. cloacae*, *K. oxytoca*, and *K. pneumoniae*, induced metabolic changes resembling that reported for PC (p<0.05) (**Figure 1B**; **Supplementary Figure S2C**), as identified through the metabolite set enrichment analysis (MSEA) conducted using MetaboAnalyst 5.0 by an over-representation analysis of disease-associated blood metabolite set, which concur with notions reported earlier in clinical metabolomics studies (30, 31).

Considering that the isolated IPMN strains were predominantly facultative anaerobes and hypoxia is a hallmark of malignant tumor growth (3), we characterized these strains using a 3D spheroid model under varying oxygen conditions, including hypoxia (1% O2) and normoxia (21% O2). Spheroids composed of hTERT-HPNE cells (immortalized normal pancreatic ductal epithelial cells) were subjected to image acquisition and analysis to evaluate the bacterial invasion efficiency and potential DNA damage outcomes (**Supplementary Figure 2**). Our results in **Figures 1C, D** present the hTERT-HPNE spheroids in a 3-dimensional assessment following bacterial exposure (MOI 1) at 18 h post-exposure, with consistent data from three independent experiments. The data revealed that, under normoxic conditions, *G. adiacens* (case H1) and *E. cloacae* (case H2) exhibited clear penetration through the spheroids, with *G. adiacens* (case H1) inducing significant DNA damage, as indicated by γH2A.X formation (**Figures 1D, E**). Under hypoxic conditions, it was observed that several more strains could adhere to and invade the hTERT-HPNE spheroids, including *G. adiacens* (case H1), *E. cloacae* (case H2), *E. faecium* (case H2), and E*. faecalis* (case C1) (**Figures 1D, F**). Hypoxic conditions also promoted significant DNA damage in *G. adiacens*-treated spheroids, with minimal damage observed in control cells (**Figures 1E, F**). And in both models, the overall cytotoxicity was around 25% with little variation between the strains (data not shown). Our data suggest that hypoxia may enhance the cellular susceptibility to the bacteria insult in the 3D sphere context of pancreatic cells, as confirmed by the γH2A.X marker protein earlier used as a proxy to detect oncogenic insult induced by other genotoxic bacteria (32). Correlation analysis further indicated a strong link between bacterial invasion and DNA damage in pancreatic cells under both hypoxic and normoxic conditions (**Figure 1G**). Collectively, these findings demonstrate that selected tumor bacteria from IPMN can adhere to and invade healthy pancreatic hTERT-HPNE spheroids under normoxic conditions, while hypoxia enables additional bacterial strains to invade. Regardless of oxygen levels, the invasiveness of bacteria strongly correlates with the extent of double-stranded DNA damage in host pancreatic cells.

### 3.2 Pancreatic cancer PANC1-heterospheroids in hypoxic environment is more sensitive to bacterial invasion and DNA damage

**Figure 2:**
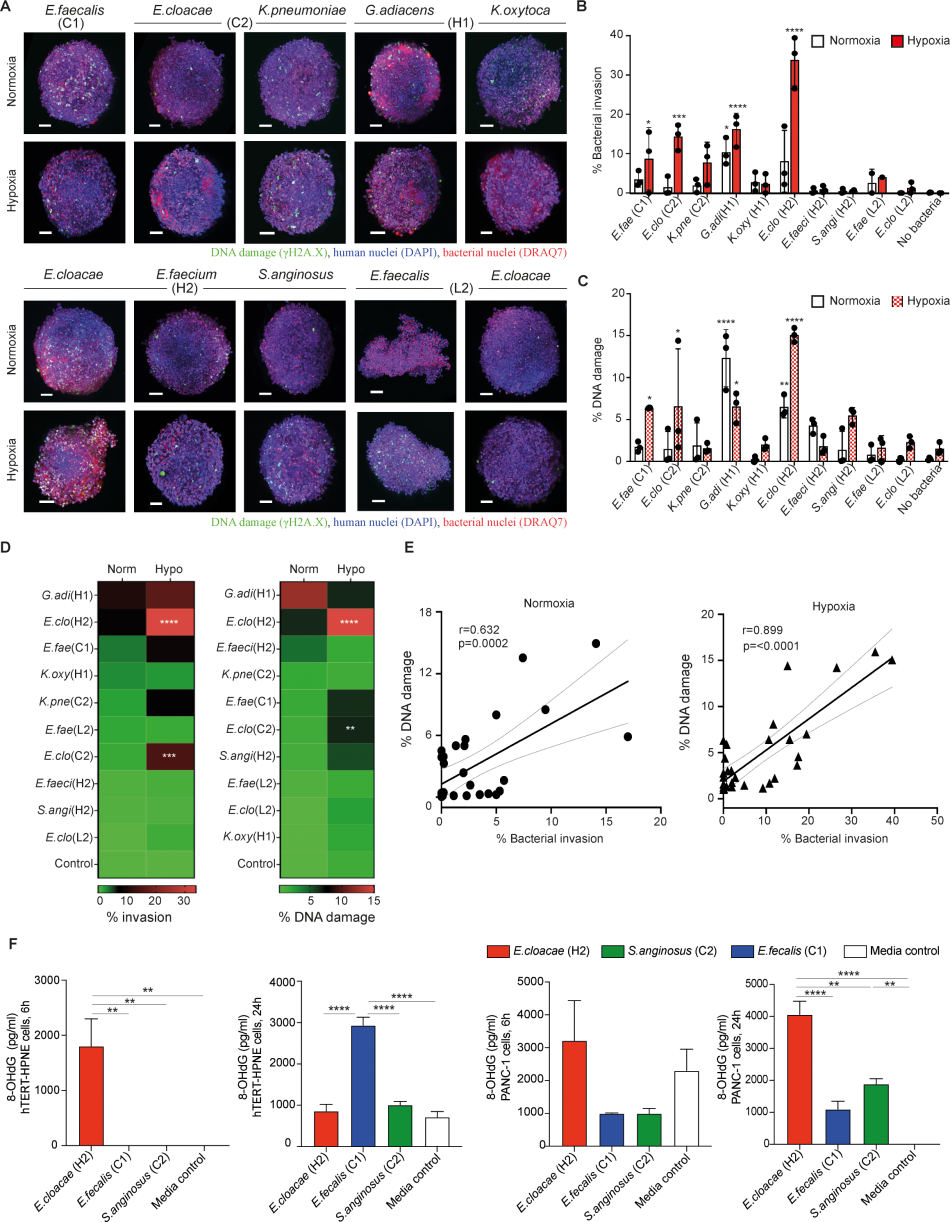
*Bacteria derived from IPMN tumors penetrate and induce DNA damage in pancreatic cancer PANC1-heterospheroids*. **(A)** PANC1-heterospheroids were cultured with or without the indicated bacterial strains as given in **Figure 1** under either normoxic or hypoxic condition (MOI 1) for 18 (h) Stained spheroids are visualized by the 3D light sheet imaging to measure phosphorylated γH2A.X formation (green, DNA damage), human nuclei (blue, DAPI), human and bacterial nuclei (red, DRAQ7). Scale bar = 100mm. **(B, C)** Summary of three experimental repeats of quantification of **(B)** bacterial invasion or **(C)** bacterial-associated DNA damage in PANC1-heterospheroids under normoxia condition (white bars) or hypoxia condition (red bars). **(D)** Level of invasiveness of bacterial strains from high to low and significant differences between normoxia and hypoxia are indicated. **(E)** Correlation between bacterial invasion and DNA damage under normoxia or hypoxia. **(F)** Levels of oxidative DNA damage expressed as concentration 8-hydroxy-2’-deoxyguanosine (8-OHdG), measured at 6 and 24 hours after exposure to indicated tumor bacteria strain in the hypoxia condition (MOI 1), in hTERT-HPNE (left panel) respectively PANC-1 (right panel) cell lines. Two way-ANOVA was used for group comparisons. *p < 0.05, **p < 0.01, ***p < 0.001, ****p < 0.0001.

Pancreatic ductal adenocarcinoma (PDAC) is characterized by dense desmoplastic stroma consisting of excessive extracellular matrix (ECM) deposition, including cancer-associated fibroblasts (CAFs), which can affect drug access and therapy response (33). Neither AsPC-1 or Capan-1 cells could generate compact spheroids with CAFs (unpublished observation). To recreate this environment, we next conducted experiments using the established PANC1-heterospheroid model composed of human PDAC cancer cells (PANC1) and CAFs of murine pancreatic stellate cells (mPSCs) as stromal components (25). Assessment of the aforementioned bacterial strains was performed using the PANC1-heterospheroid model under hypoxic or normoxic conditions (**Figure 2A**). Again, we observed significant invasion and DNA damage caused by *G. adiacens* (case H1) and *E. cloacae* (case H2) in this cancer spheroid model under normoxic conditions (**Figures 2B, C**). The hypoxic condition allowed several more strains, including *E. cloacae* (case C1), *E. faecalis* (case C1), *K. pneumoniae* (case C2), *G. adiacens* (case H1), and *E. cloacae* (case H2), to invade the cancer spheroids (**Figure 2D**). Notably, significant DNA damage was detected in the cancer spheroids exposed to *G. adiacens* (case H1), *E. cloacae* (case H2), *E. cloacae* (case C1), and *E. faecalis* (case C1) compared to that in the control cells 18 h after incubation (**Figure 2C**). Similar to earlier observations in hTERT-HPNE spheroids (**Figure 1D**), DNA damage of γH2A.X formation was strongly correlated with bacterial invasion in PANC1-heterospheroids (**Figure 2E**). In line with these notions, increasing levels of oxidized guanine species (**Figure 2F**, **Supplementary Figure 4**) were also detected in both hTERT-HPNE- (left panel) and PANC-1 cells (right panel) after exposing to the highly pathogenic *E. cloacae* (case H2), *E. cloacae* (case C1), and not to *S. anginosus*, seen at 6 or 24 hours post-exposure with some variation between the cell types. Considering the possibility that hypoxia may introduce damage to the pancreatic cells without bacterial insult. Having done further experiment to address this (**Supplementary Figures 5A, B**), we found that hypoxia by itself causes no significant change to neither cell proliferation (spheroid size), cell death (% of dead cells) or DNA damage (gH2A.X phosphorylation). The only effect we observed was an increase in the side scatter value in the flow cytometric analysis, which indicate an increased granularity possibly due to cell stress. Taken together, our findings demonstrate that specific strains of tumor bacteria can penetrate and invade pancreatic spheroids, and their invasiveness is further augmented under hypoxic conditions, particularly in strains isolated from cancer cases (cases C1 and C2) (**Figure 1D**).

### 3.3 The secretome of tumor bacteria exhibiting riboflavin biosynthesis competence activates human MAIT cells

**Figure 3:**
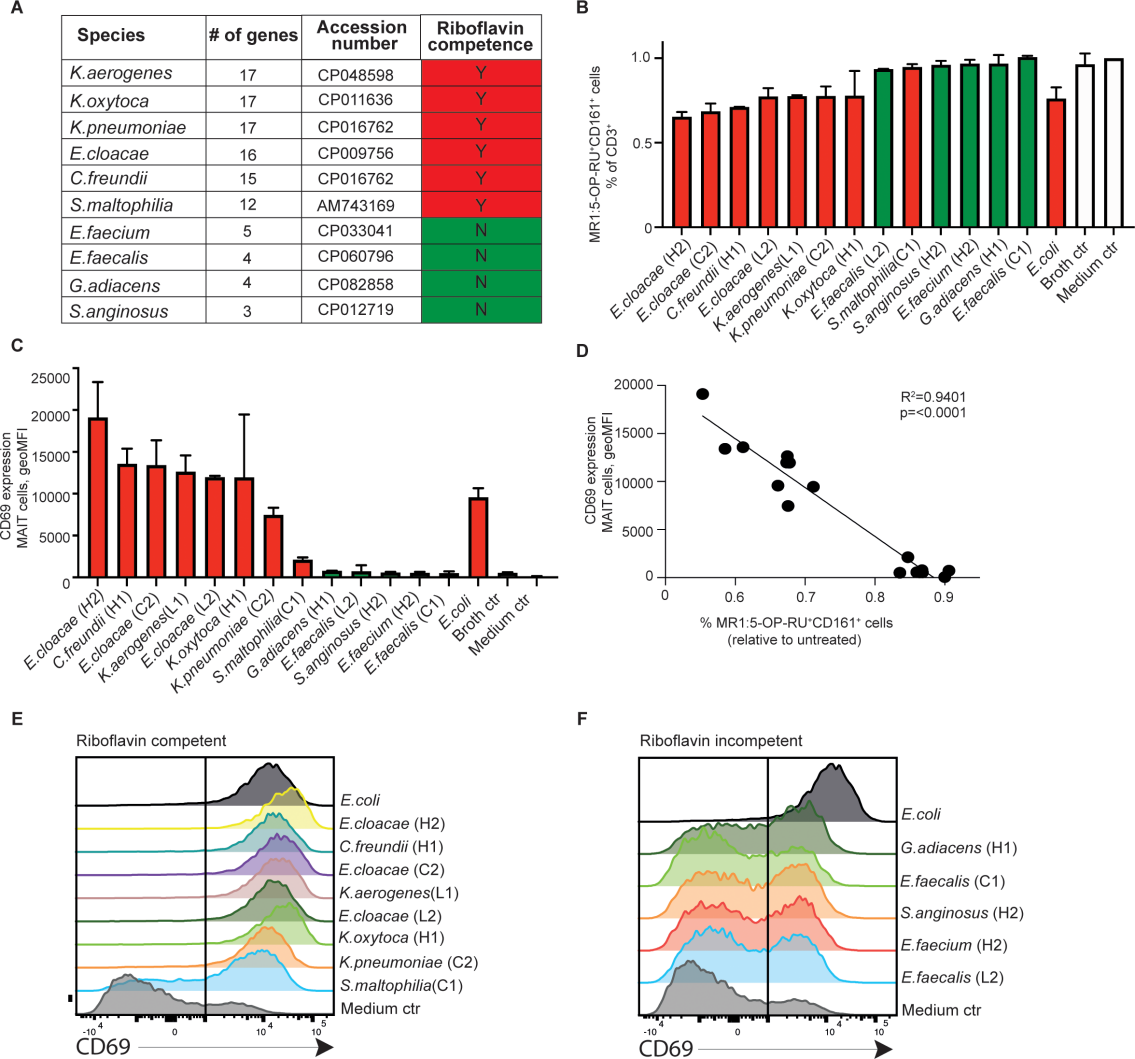
Riboflavin-competent tumor bacterial isolates activate blood human MAIT cells. **(A)** KEGG pathway prediction of riboflavin competence in bacteria strains isolated from the pancreatic cystic tumors. Presence (red) or absence (green) of riboflavin competence is shown. Bacterial secretome-induced MAIT cell activation as indicated by **(B)** TCR downregulation in MR1:5-OP-RU+ and CD161-gated MAIT cell population, and **(C)** surface CD69 expression (geometric mean fluorescence intensity) in MAIT cells of peripheral blood mononuclear cells (PBMC) by indicated bacteria strain after 24 h of exposure. **(D)** Linear regression analysis on surface CD69 expression levels in gated MAIT cells and relative percentage of MR1:5-OP-RU-binding cells in the CD3+ lymphocyte population as shown in **(B, C)**. **(E, F)** Flow cytometry histograms in the summary plots showing the CD69 expression after stimuli with indicated riboflavin competent strains **(E)** or riboflavin non-competent strains **(F)**, and medium control (ctrl) at 24 hours after stimulation.

PC tumor tissues exhibit variable presence of non-conventional MAIT cells within the immune cell compartment (34). Although MAIT cells are known to recognize microbial metabolites, specifically from the riboflavin biosynthesis pathway, how they respond to patient tumor-derived bacteria has not been reported. By cross-referencing against the KEGG database and reference genomes, our analysis showed that *Klebsiella* spp., *E. cloacae*, *Citrobacter freundii*, and *Stenotrophomonas maltophilia* may possess a complete set of genes required for riboflavin synthesis, whereas other isolates, including Gram-positive strains *Enterococcus* spp., *G. adiacens*, and *S. anginosus*, lacked this biological pathway (**Figure 3A**).

To test the responsiveness of MAIT cells to microbial metabolism in our tumor bacterial strains, we first exposed peripheral blood mononuclear cells (PBMCs) from healthy donors to bacterial culture supernatants that had undergone double-sterile filtration. Utilizing CD161 and MR1:5-OP-RU tetramer gating on the CD3+ lymphocyte population, we observed that upon overnight supernatant exposure, a considerable MAIT cell population displayed upregulation of CD69 (**Figures 3C-F**), accompanied by a concurrent activation-induced Vα7.2 TCR downregulation, as defined by the percentage of cells binding MR1:5-OP-RU tetramer in the total CD3+ population (R2 = 0.94, p<0.0001; **Figures 3B, D**). We did not detect any human cytokines such as IL-2 and IL-12 (MAIT cell activators) in the baseline culture supernatant (data not shown). Therefore, our present finding supports that the MAIT cell-reactive 5-OP-RU riboflavin derivative represent the main activator in select bacterial secretome, in line with that predicted by our riboflavin competency analysis (red). Consistent activation of MAIT cell CD69 was observed across the anticipated isolates, with *E. cloacae* from a high-grade dysplasia case (case H2) being the most potent activator. The activation level was comparable to that of the commonly used laboratory control strain, *Escherichia coli*. Conversely, strains predicted to lack riboflavin synthesis capabilities (green) failed to trigger MAIT activation (**Figures 3E, F**). Our findings highlight the specific interaction between MAIT cells’ semi-variant Vα7.2 TCR and the microbial 5-OP-RU ligand exclusively drives MAIT cell recognition of pancreatic tumor-derived bacteria. Consequently, our observations strongly indicated that peripheral MAIT cells can detect and react to bacterial species from IPMN neoplastic development via riboflavin-derived metabolite sensing.

### 3.4 MAIT cells restrict extra- as well as intracellular bacterial invasion in pancreas spheroids

**Figure 4:**
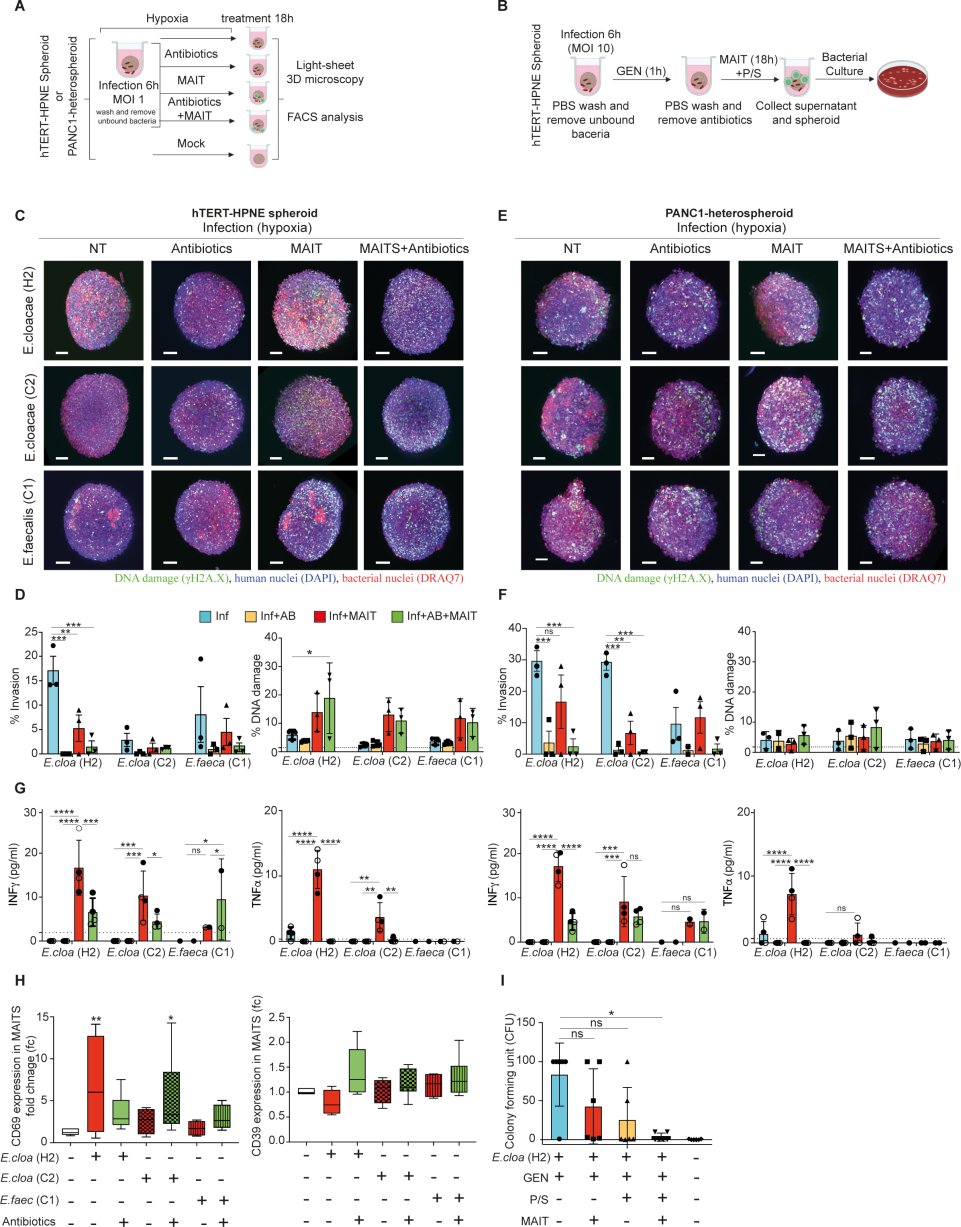
MAIT cell inhibition of riboflavin competent bacteria in pancreatic spheroids under hypoxic conditions. **(A, B)** Schematic representation of the extracellular and intracellular infection experimental design for the result in **Figure 4**. The hTERT-HPNE **(C)** or the PANC1-heterospheroid **(E)** were cultured in hypoxia with or without *E. cloacae* (H2), *E. cloacae* (C2), or *E. faecalis* (C1) at MOI 1 for 6 (h) Thereafter expanded human donor MAIT cells (20,000 cells) or antibiotic (Normocin,100mg/mL) were added for additional 18 hours and fixed. Spheroids were then evaluated by 3D imaging in light sheet microscopy for measurement of phosphorylated γH2A.X formation (green), human nuclei DAPI staining (blue), or human and bacterial nuclei in DRAQ7 (red). Scale bar (100mm). NT=Not treated. Summary of the levels of bacterial invasion and DNA damage in **(D)** hTERT-HPNE spheroids or **(F)** PANC1-heterospheroids18 hours after bacteria exposure from three independent experiments, with expanded MAIT cell preparations from three individual healthy donors. Line in DNA damage indicates background signal. **(G)** Cytokine analysis of MAIT cell co-cultured with and without bacteria-exposed (MOI 1) or/and antibiotic treated hTERT-HPNE (left panel) or the PANC1-heterospheroid (right panel), n=3 for each condition. Two way-ANOVA was used for group comparisons. *p < 0.05, **p < 0.01, ***p < 0.001, ****p < 0.0001. **(H)** Surface CD69 and CD39 expression (fold change *vs*. unexposed) in expanded MAIT cells in response to bacteria-exposed hTERT-HPNE spheroids. Significance was determined using pair-wise comparisons. Wilcoxon signed-rank test was used for comparison of dependent groups. *p<0.05, **p<0.01, ***p<0.001, ****p<0.0001, ns = not significant. **(I)** Intracellular bacterial infection in hTERT-HPNE spheroids was established in hypoxia incubator with *E. cloacae* (H2, MOI 10) and Gentamicin (GEN, 0.3 mg/mL, 1h), then incubated for another 18 h with either Penicillin-Streptomycin (P/S) or expanded MAIT cells (MAIT), or combination of both in hypoxia incubator. Levels of the intracellular hiding bacteria in lysed cell pellet for indicated condition are given as Colony Forming Unit (CFU). GEN = gentamicin. *p<0.05, ns, not significant.

We next investigated the antibacterial effect of MAIT on bacterial invasion in pancreatic spheroids by applying high-purity *ex vivo* expanded human MAIT cells (**Supplementary Figure 1**), which we recently developed for immunotherapeutic applications aimed at improving cancer immunotherapy for pathogen-associated solid tumors (19, 20). The hTERT-HPNE monospheroids (healthy pancreatic cells) or cancerous PANC1/mPSC-heterospheroids (pancreatic epithelioid carcinoma/stellate cells) were grown and inoculated under hypoxic condition with *E. cloacae* (cases H2 and C2) or *E. faecalis* (case C1). Following a 6h incubation to permit maximal target binding, attachment and entry into human spheroid configured-target, non-adherent bacteria were washed and removed (35). The spheroids were thereafter treated with either the broad-spectrum antibiotic preparation Normocin, MAIT cells, or a combination of both (**Figure 4A**). As shown in **Figures 4C, D**, as Normocin effectively restricted the bacterial invasion in hTERT-HPNE spheroids, MAIT cells or the combined MAIT cell and normocin treatment also restricted *E. cloacae* (case H2) infiltration of the whole spheroid in three independent spheroid experiments. Despite reduced bacterial invasion, the cells in these co-cultures exhibited DNA damage potentially resulting from the cytolytic effects of MAIT cell activation and inflammatory cytokines. As the outcome was associated with significant secretion of IFNg and TNFa and upregulation of CD69 expression in the co-cultured MAIT cells (**Figures 4G, H** left panel), but not CD39 upregulation and was connected to activated-increased cell death (data not shown). Adding Normocin could dampen bacteria induced MAIT cell activation in *E. cloacae* (case H2) but not *E. cloacae* (case C2). On the other hand, *E. cloacae* (case C2) appeared low invasive and mildly activated MAIT cells. A similar result was produced by *E. faecalis* (C1), which is presumed to lack riboflavin competence based on KEGG analysis (**Figures 4D, H**), suggesting that active bacterial riboflavin metabolism is required to activate MAIT cells in infected hTERT-HPNE pancreatic spheroids.

In the cancerous PANC1/mPSC-heterospheroids (**Figures 4E–G** right panel), all three treatment types appeared to restrict the invasion of *E. cloacae* (H1 and C2), including MAIT cells, which showed a partial effect. This was not observed in riboflavin-incompetent *E. faecalis*. Low levels of cellular DNA damage were also observed in these cultures.

Because intracellular survival may serve as a microbial feature to escape antimicrobial chemotherapy (9), we examined whether MAIT cells were effective against intracellular bacteria present in spheroids using a gentamicin (GEN) survival assay (**Figure 4B**). In this assay, we focused on the *E. cloacae* (H1), which was confirmed to be gentamicin-susceptible (MIC ≤ 0.25 mg/L, below the breakpoint of 2 mg/L as determined by EUCAST Disk Diffusion Testing) and lacks the gentamicin heteroresistance cpxRA system (36), as confirmed by whole genome sequencing (unpublished result). Spheroids infected with *E. cloacae* (case H2) were maintained and studied under hypoxic conditions. While GEN treatment effectively eliminated extracellular bacteria, residual intracellular infection was detected when the cells were lysed and tested for bacterial growth (**Figure 4I**). We found that intracellular-hiding *E. cloacae* was variably sensitive to the addition of MAIT cells or penicillin-streptomycin (P/S). Interestingly, a significant synergistic contribution from the latter two treatments in combination was found to effectively eliminate intracellular bacterial invasion.

Consequently, our results indicate that MAIT cells can restrict both extracellular and intracellular invasion of pancreatic spheroids caused by IPMN-derived tumor bacteria in a hypoxic physiological environment.

## 4 Discussion

Polymorphic microbes have emerged as a new hallmark of cancer (3). While much research has focused on the intestinal microbiota, the recent discovery of a local microbiome within tumors prompted our investigation of clinical bacterial strains isolated from pancreatic IPMN tumors (9). Our findings in 3D pancreatic spheroids demonstrate that patient-derived tumor bacteria from IPMN exhibit varying degrees of pathogenicity and can induce oncometabolic disturbances in different pancreatic cell types. These results are consistent with our previous findings in conventional 2D cell cultures (9). Furthermore, our functional screening of the IPMN microbiota highlighted that the pathogenicity of facultative metabolism is influenced by environmental factors, particularly oxygen levels. Additionally, the essential metabolism of these bacteria is a potential target for immune-mediated clearance. Importantly, the heterogeneity of the cellular pathology induced by individual IPMN bacteria isolated from patients expands upon what has been reported with traditional laboratory strains. Our use of light-sheet microscopy to examine patient strains and spheroids provided a better understanding of 3D cellular invasion and DNA damage processes compared to conventional 2D microscopy.

Several clinically relevant observations have been made based on our results. As for the association of metabolomic changes with carcinogenesis, some of the metabolites we have identified as upregulated in infection with some of these tumor bacteria are reported to be specific to PC. These include 2-hydroxyglutarate, induced by several clinical isolates here, has been associated with metastatic formation and immune evasion by PC (37) and a marker for PC risk (38). Putrescine- and ornithine induction, by multiple bacterial strains found here, not only are promoted by oncogenic KRAS activation (39) but also are proposed as PC biomarker (40). Furthermore, the metabolites induced by gammaproteobacterial strains in our experimental infections, particularly polyamines such as cadaverine and putrescine, were previously found to be enriched in IPMN cystic tumors classified as high-grade dysplasia (HGD) or cancer. These metabolites formed a cluster association with *Enterobacteriacea*, *Granulicatella*, *Klebsiella* in an integrated IPMN metabolome and microbiome analysis that was performed on independent clinical pancreatic cyst fluid material (30). The Krebs cycle dicarboxylate metabolites, including succinate, fumarate, and malate, detected in IPMN cysts were also able to differentiate malignant from benign IPMN tumors. Therefore, the metabolic changes induced by tumor-associated microbiota in pancreatic cells may contribute to the reported pancreatic cancer biomarkers, as indicated by our MetaboAnalyst 5.0 overrepresentation analysis. Another clinically relevant finding from our data is that *G. adiacens*, *E. cloacae*, and *E. faecalis* isolated from IPMN cases associated with HGD or cancer exhibited higher invasiveness in pancreatic spheroids than *E. cloacae* and *E. faecalis* strains isolated from low-grade dysplasia tumor samples. Genetic differences between bacterial strains from different diagnostic conditions are currently being examined will provide more insight, and metabolites such as succinate that can be produced by bacteria alone (41) could also have impacted our result.

Bacteria are susceptible to pathogenic changes under environmental stress (42) and hypoxia is a prominent hallmark of cancer (3). Thus, our study provides valuable insights into bacterial adaptation in the tumor microenvironment. Our spheroid results demonstrated that the strains with increased pathogenicity under hypoxia were *E. cloacae* and *E. faecalis* isolates obtained from IPMNs with confirmed advanced cancer. While we used the phosphorylated histone γH2A.X marker to detect DNA damage caused by pks+ Escherichia coli genotoxins (18), the possibility of *pks*+ islet expression in our strains or the presence of other genotoxins, including cytolethal distending toxin, tilimycin, and indolimines, cannot be ruled out (43). Whether the bacteria invasion into the tumoroids causes other effects other than DNA damage is relevant to investigate.

Other tumorigenic mechanisms may involve inhibition of p53 tumor suppressor activity by enterobacterial lipopolysaccharides (44), underscoring the importance of MAIT cell capacity to specifically target these bacteria. Such subcellular- and/or strain-specific factors (43, 45) that may confound observations noted on the invasiveness and DNA damaging property in for instance *E. cloacae* (C2), or on the MAIT cell activation cannot be excluded in this study.

Antibiotic treatment had a limited clinical effect on the IPMN microbiota, as all isolates were derived from patients who had received antibiotics prior to surgery (9). Factors such as bioavailability, therapeutic levels, and antimicrobial resistance mechanisms may contribute to this limitation (46). Our notion that antibiotics could not entirely prevent the metabolic alterations in pancreatic cells after the bacterial exposure, suggest that microbial attachment and entry alone may also contribute to low level of cellular activation signals and cellular insult. Details in the adhesion and invasion mechanisms employed by tumor bacteria are crucial for the development of potential clinically relevant treatments. Immune-mediated blockade, on the other hand, against microbial adhesins to human target cells could involve specific antibodies or nanobodies for infusion or vaccination, but competitions from existing endogenous antibodies could pose a challenge (47). On the other hand, the MAIT cell approach that we investigate here targets the essential metabolites of pathogenic microbes and holds great promise for addressing the challenges posed by microbiota-bearing tumors. MAIT cells are well known for their antimicrobial functions and protective role against infectious pathogens, including fungi that may also hide in tumors (13, 14). In addition, MAIT cells have intrinsic cytotoxic potential, effector memory characteristics, and a broad repertoire of tissue-homing chemokine receptors that are highly relevant for cancer immunotherapy to combat solid tumors (19, 20). To our knowledge, this is the first study to test MAIT cells against patient-derived tumor bacteria in a 3D hypoxic context. Our results provide clear evidence that MAIT cells can recognize patient-derived IPMN bacteria with known riboflavin competence, leading to a significant reduction in invasion rate, even in a tumor-like spheroid environment. The observed upregulation of CD69 and its inverse correlation with MAIT cell TCR downregulation support the activation of MAIT cells via their TCR, which is necessary for subsequent cytokine responses and target killing (48). Notably, MAIT cell-recognized microbes typically include Gram-negative bacteria, which are often associated with unfavorable outcomes during anti-PD-1 treatment of melanomas (49).

Our study has some limitations; it focused on microbiota strains derived from IPMN and did not encompass the full spectrum of known tumor-resident microbiota, such as fungi, which are also recognized by MAIT cells. While our findings provide evidence that MAIT cells can recognize both extracellular and intracellular bacteria, even in the presence of antibiotic treatment, it should be noted that their recognition was specific to riboflavin-competent microorganisms. A more detailed visualization of bacterial invasion, such as to distinguish cellular invasion from attachment using high-resolution microscopy or image-stream FACS, could have provided additional insights. Furthermore, evaluations were limited to short-term follow-ups due to typical short lifespan of complex cellular infection models (35). Additionally, few different types from the classical progenitor (Capan-2 and AsPC-1), epithelial (HPNE), and basal-like squamous (PANC-2) pancreatic cell groups were studied; future studies on a broader comparative analysis on more classical- *vs*. basal-like PDAC subtypes (50) shall give more insight. In the heterospheroid model, the presence of mammalian cells from two species hindered the analysis of MAIT cell responses. Still, we were able to successfully demonstrate MAIT cell responses to non-cancer spheroids derived from normal pancreatic cells.

Considering the global concern regarding bacterial resistance to antibacterial drugs, it is possible that human MAIT cells could contribute to combating the tumor-resident microbiota. In addition to their ability to directly kill tumor cells and orchestrate downstream immune responses, including the control of integrated oncoviruses (19, 20), MAIT cells also possess the capacity to control antimicrobial resistance (51). Our study opens a new direction for targeting tumor-associated microbiota by leveraging their essential metabolic pathways. The concept of utilizing MAIT cells for managing pathogenic microbiota in tumoral diseases deserves further exploration to enhance the existing treatment options.

## Data Availability

The datasets presented in this study can be found in online repositories. The names of the repository/repositories and accession number(s) can be found below: https://www.ebi.ac.uk/biostudies/, S-BSST1747.
