# Supplementary figures and images for: Patient-derived pancreatic tumor bacteria exhibit oncogenic properties and are recognized by MAIT cells in tumor spheroids
Source: Front Immunol. 2025 Apr 22;16:1553034. doi: 10.3389/fimmu.2025.1553034 (PMC12053177; doi:10.3389/fimmu.2025.1553034)

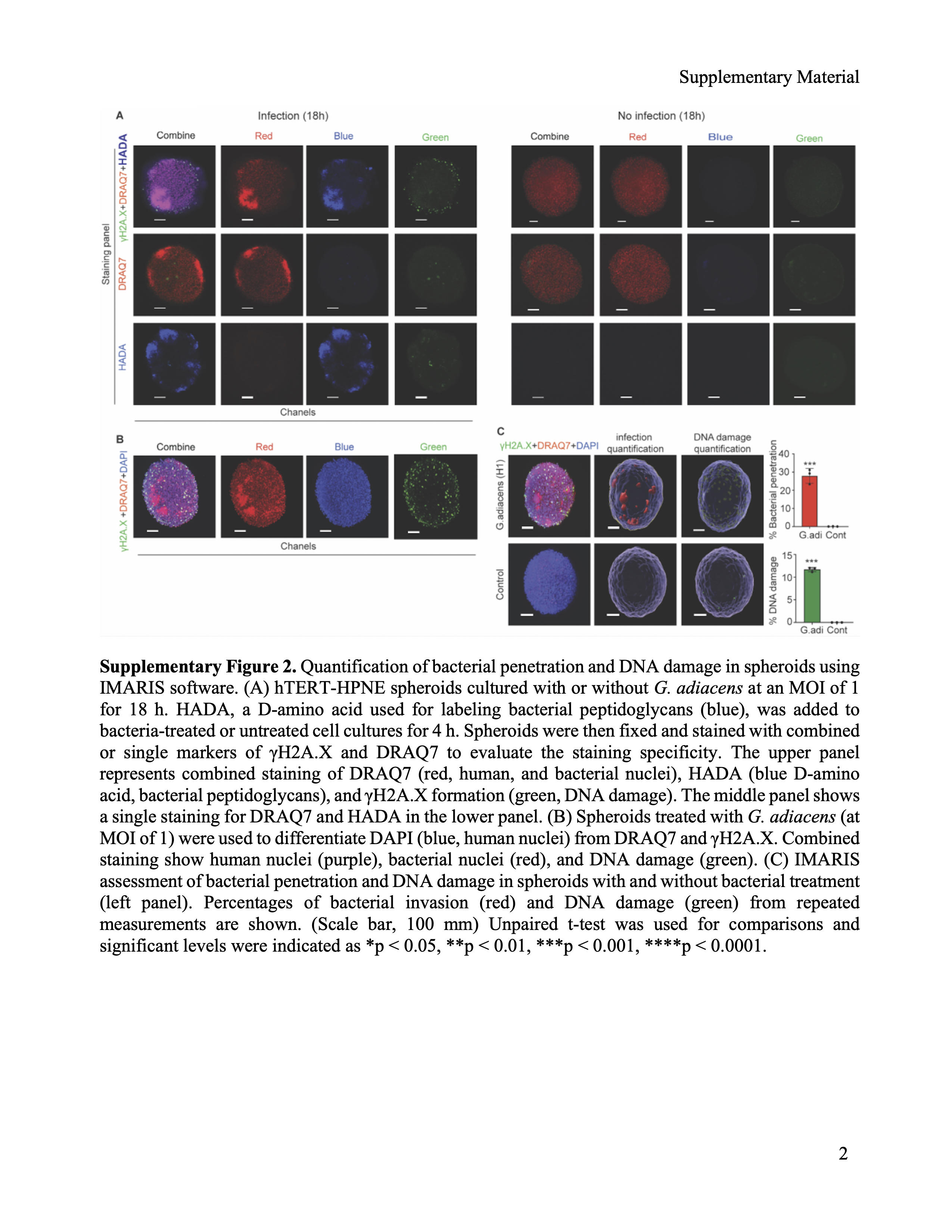

Supplement: Supplementary file 2 [file Image2.jpeg]

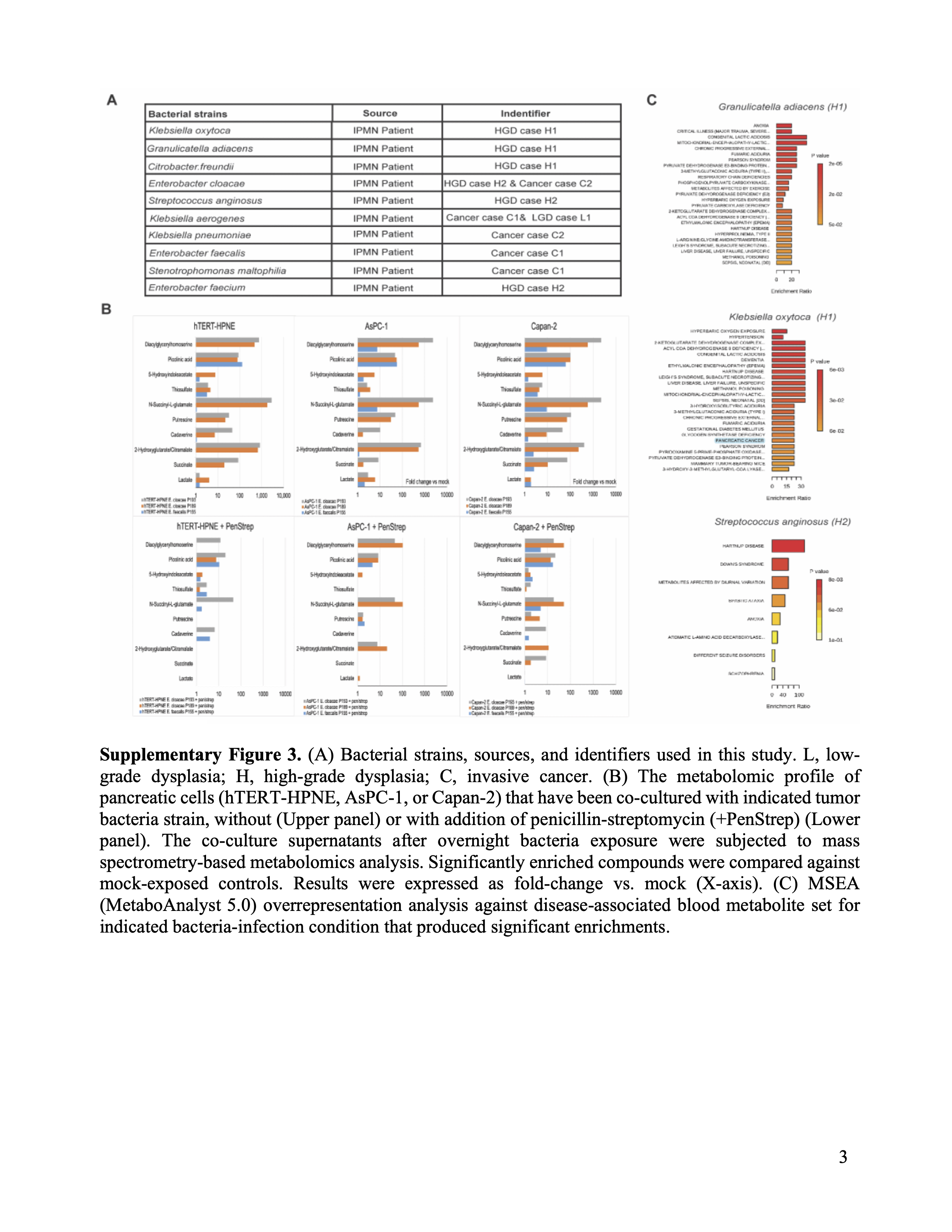

Supplement: Supplementary file 3 [file Image3.jpeg]

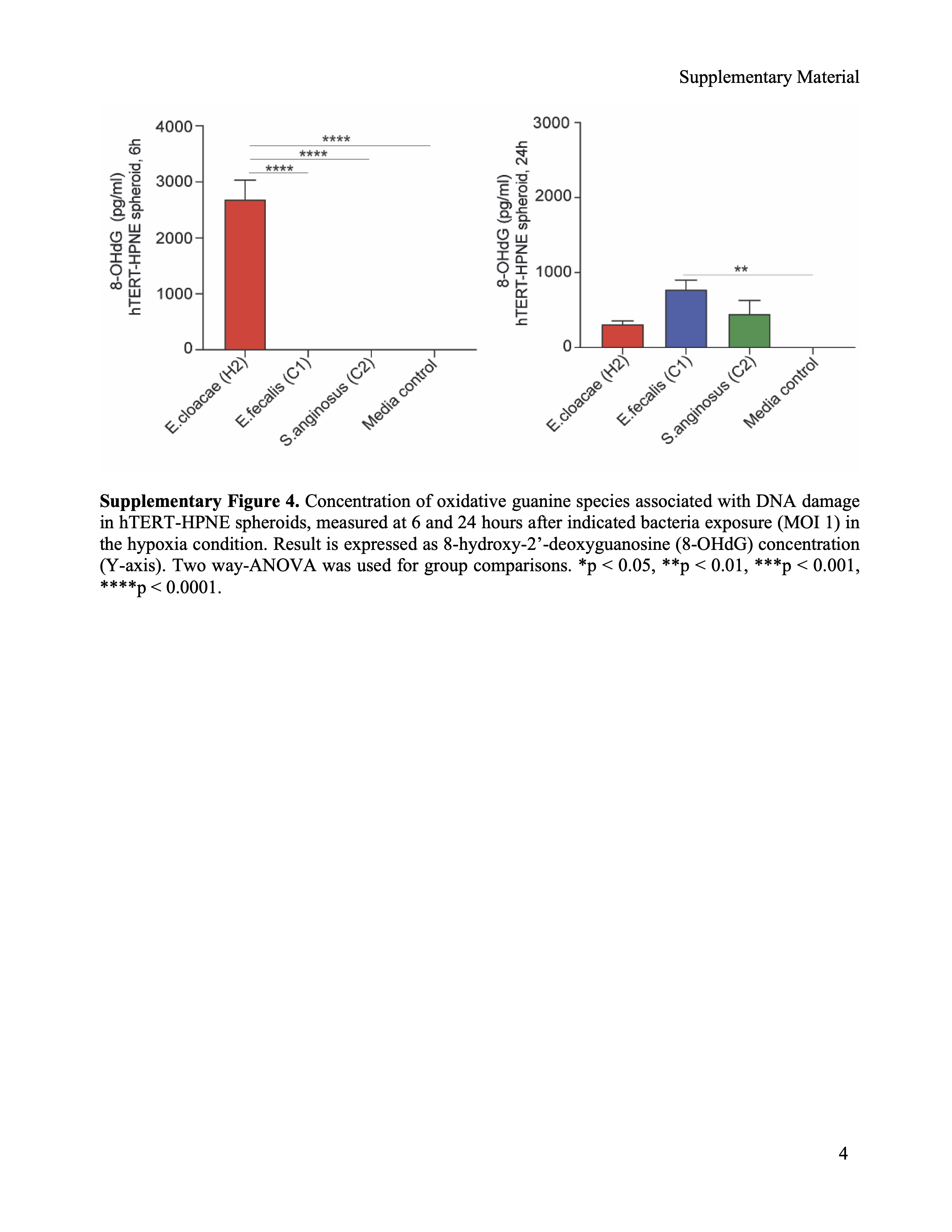

Supplement: Supplementary file 4 [file Image4.jpeg]

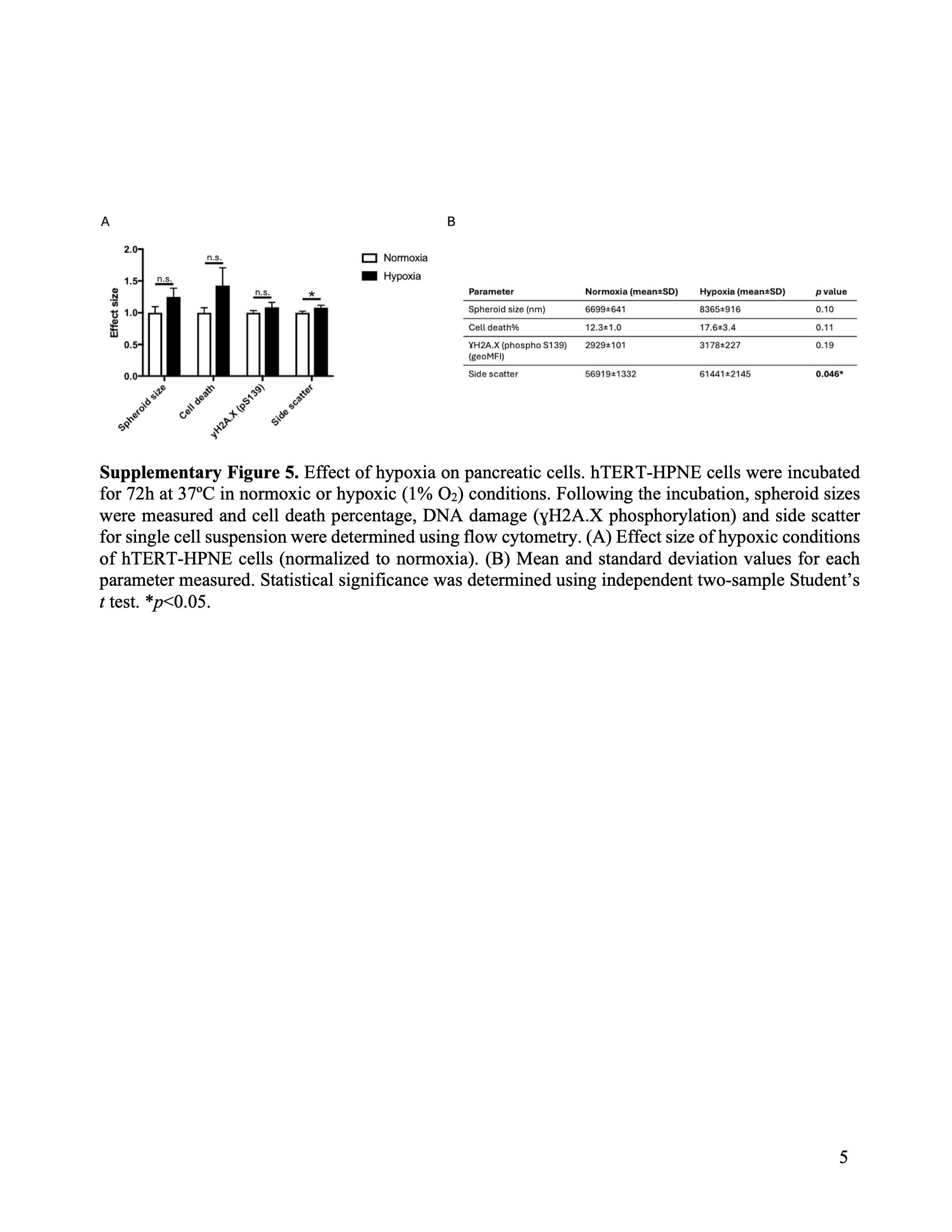

Supplement: Supplementary file 5 [file Image5.tiff]
